# AURORA: bariatric surgery registration in women of reproductive age - a multicenter prospective cohort study

**DOI:** 10.1186/s12884-016-0992-y

**Published:** 2016-07-29

**Authors:** Goele Jans, Christophe Matthys, Sarah Bel, Lieveke Ameye, Matthias Lannoo, Bart Van der Schueren, Bruno Dillemans, Luc Lemmens, Jean-Pierre Saey, Yves van Nieuwenhove, Pascale Grandjean, Ben De Becker, Hilde Logghe, Marc Coppens, Kristien Roelens, Anne Loccufier, Johan Verhaeghe, Roland Devlieger

**Affiliations:** 1 Department of Development and Regeneration, KU Leuven, Herestraat 49, 3000 Leuven, Belgium; 2 Department of Clinical and Experimental Medicine, KU Leuven, Herestraat 49, 3000 Leuven, Belgium; 3 Department of Endocrinology, University Hospitals Leuven, Herestraat 49, 3000 Leuven, Belgium; 4 Scientific Institute of Public Health, Department of Public Health and Surveillance, Unit Surveys, Lifestyle and Chronic Diseases, Juliette Wytsmanstraat 14, 1050 Brussels, Belgium; 5 Department of Abdominal Surgery, University Hospitals Leuven, Herestraat 49, 3000 Leuven, Belgium; 6 Department of Abdominal Surgery, St-Jan Hospital Bruges, Ruddershove 10, 8000 Bruges, Belgium; 7 Department of Abdominal Surgery, St-Nikolaas Hospital, Moerlandstraat 1, 9100 St-Niklaas, Belgium; 8 Medicosurgical unit for metabolic diseases, CHR Mons Hainaut, 5 avenue Baudouin de Constantinople, 7000 Mons, Belgium; 9 Department of Gastrointestinal Surgery, Ghent University Hospital, De Pintelaan 185, 9000 Ghent, Belgium; 10 Department of Obstetrics and Gynecology, CHR Mons Hainaut, 5 avenue Baudouin de Constantinople, 7000 Mons, Belgium; 11 Department of Obstetrics, Gynecology and Reproduction, St-Augustinus Hospital Wilrijk, Oosterveldlaan 24, 2610 Wilrijk, Belgium; 12 Department of Obstetrics and Gynecology, St-Lucas Hospital Bruges, St-Lucaslaan 29, 8310 Bruges, Belgium; 13 Department of Obstetrics and Gynecology, ZNA Middelheim, Lindendreef 1, 2020 Antwerp, Belgium; 14 Department of Obstetrics and Gynecology, Ghent University Hospital, De Pintelaan 185, 9000 Ghent, Belgium; 15 Department of Obstetrics and Gynecology, St-Jan Hospital Bruges, Ruddershove 10, 8000 Bruges, Belgium; 16 Department of Obstetrics and Gynecology, University Hospitals Leuven, Herestraat 49, 3000 Leuven, Belgium

**Keywords:** Obesity, Bariatric surgery, Reproduction, Fertility, Contraception, Pregnancy, Breast feeding

## Abstract

**Background:**

The expansion of the obesity epidemic is accompanied with an increase in bariatric procedures, in particular in women of reproductive age. The weight loss induced by the surgery is believed to reverse the negative impact of overweight and obesity on female reproduction, however, research is limited to in particular retrospective cohort studies and a growing number of small case-series and case-(control) studies.

**Methods/design:**

AURORA is a multicenter prospective cohort study. The main objective is to collect long-term data on reproductive outcomes before and after bariatric surgery and in a subsequent pregnancy. Women aged 18–45 years are invited to participate at 4 possible inclusion moments: 1) before surgery, 2) after surgery, 3) before 15 weeks of pregnancy and 4) in the immediate postpartum period (day 3–4). Depending on the time of inclusion, data are collected before surgery (T1), 3 weeks and 3, 6, 12 or x months after surgery (T2-T5) and during the first, second and third trimester of pregnancy (T6-T8), at delivery (T9) and 6 weeks and 6 months after delivery (T10-T11). Online questionnaires are send on the different measuring moments. Data are collected on contraception, menstrual cycle, sexuality, intention of becoming pregnant, diet, physical activity, lifestyle, psycho-social characteristics and dietary supplement intake. Fasting blood samples determine levels of vitamin A, D, E, K, B-1, B-12 and folate, albumin, total protein, coagulation parameters, magnesium, calcium, zinc and glucose. Participants are weighted every measuring moment. Fetal ultrasounds and pregnancy course and complications are reported every trimester of pregnancy. Breastfeeding is recorded and breast milk composition in the postpartum period is studied.

**Discussion:**

AURORA is a multicenter prospective cohort study extensively monitoring women before undergoing bariatric surgery until a subsequent pregnancy and postpartum period.

**Trial registration:**

Retrospectively registered (July 2015 - NCT02515214)

## Background

Obesity among women aged 20 years or older has become a global health problem. The World Health Organization estimated that about 13 % of the world’s adult female population was obese in 2014 [1]. It is demonstrated that obesity negatively affects reproduction, illustrated by increased fertility problems by anovulation in women with polycystic ovary syndrome [2]. A future pregnancy in these women is accompanied with an increased risk for gestational diabetes mellitus, (pre)eclampsia, pregnancy induced hypertension, induction of labor, cesarean section and more neonates with a birth weight ≥ 4000 g [3, 4]. In addition, breastfeeding initiation and duration ratios appear to be lower in obese women [5]. Therefore, the American College of Obstetrics and Gynecology (ACOG) advices women to lose weight before conception [6]. Lifestyle modifications are put forward as the first-line treatment, however, these modifications rarely result in successful long term weight loss. Nowadays, bariatric surgery in combination with lifestyle modifications is considered to be the best treatment to achieve successful weight loss on the long term in individuals with a BMI ≥ 35 kg/m^2^ with comorbidities or a BMI ≥ 40 kg/m^2^.

The most commonly performed procedure worldwide is the Roux-en-Y gastric bypass (RYGB – 45 %) [7]. This procedure induces a combination of restriction in food intake and a certain degree of malabsorption. Procedures that mainly induce a restriction in food intake, such as sleeve gastrectomie (SG) and laparoscopic adjustable gastric banding (LAGB), are performed less frequently. SG was performed in 37 % of the patients and LAGB in 10 % of the patients worldwide in 2013 [8]. Procedures that mainly intend to induce nutrient malabsorption by bypassing the gastro-intestinal tract in a complex way, such as biliopancreatic diversion with or without duodenal switch (BPD/DS), are performed rarely (2.2 %) [7]. Bariatric surgery is found to decrease the overall, cardiovascular and diabetes related mortality, and implicates greater improvements in quality of life compared to non-surgical controls [9–11].

The increasing number of bariatric procedures in female adolescents and women of reproductive age is a major challenge for follow-up of pregnancies [12, 13]. Official organizations recommend delaying pregnancy 12 to 18 months after surgery in order to avoid the period of rapid weight loss [6]. Small studies comparing the pre- and post-bariatric surgery fertility status report an improvement in spontaneous fertility rates [14, 15]. This improved fertility makes safe and reliable contraception necessary in order to avoid early pregnancy, however, evidence on contraceptive safety and effectiveness is limited [16–18]. Concerns also rise on the potential impact of bariatric surgery on future pregnancy. Adverse obesity-related maternal, fetal and neonatal outcomes are reported less frequent in the bariatric pregnant population compared to the (morbidly) obese pregnant population, including the risk for gestational diabetes mellitus, hypertensive disorders in pregnancy, cesarean section, large-for-gestational age and +4 kg babies [16, 19–21]. A clinical challenge has risen with the diagnosis of gestational diabetes mellitus in pregnant women with previous bariatric surgery, since Freitas et al. [22] illustrated that almost 60 % of the women develops severe dumping symptoms by using the standard diagnostic methods for gestational diabetes (oral glucose tolerance test). Alternative methods, including home glucose monitoring and the assessment of hbA1c or fasten glucose levels, might be considered. Additionally, caution is needed with surgical complications and nutritional deficiencies in pregnancy, since both complications have been associated with increased perinatal mortality rates, preterm birth, small-for-gestational age and growth restricted babies and fetal and neonatal abnormalities [23, 24]. Several cases of severe surgical complications, including small bowel volvulus, obstruction and subsequent internal herniation, have been reported [25]. Recently, the incidence of internal herniation in pregnancy following bariatric surgery was estimated on 1 % in a Danish retrospective register-based cohort study [16, 26].

The nutritional status after bariatric surgery is often poor, especially in individuals with procedures inducing malabsorption by bypassing a large part of the small intestine [27]. In RYGB patients, mechanical digestion and acid secretion are limited, possibly leading to an impaired digestion and absorption of certain micronutrients (e.g. iron and vitamin B-12) and a reduction in the secretion of intrinsic factor (further complicating the absorption of certain micronutrients). Deficiencies can also be caused by insufficient intake of nutrients, food intolerance and excessive nausea and vomiting [27]. The combination of a bariatric procedure and the physiological changes during pregnancy theoretically increases the risk for nutritional deficiencies. Several case-reports and small cohort studies have been published on adverse maternal, fetal and neonatal outcomes after vit K1, vit B-12, vit A, folate and iron deficiencies [16, 28, 29]. These nutritional deficiencies, combined with poor eating habits in pregnant women with bariatric surgery [30], can affect the nutritional value of breast milk. Case reports have been published of clinical vitamin B-12 deficiencies in newborns receiving exclusively breastfeeding from vit B-12 deficient mothers with a history of bariatric surgery [31–33].

### Need for more research?

The current evidence on reproduction outcomes after bariatric surgery is mainly based on retrospective cohort studies, and a growing number of small case(-control) series. A control group is frequently missing and often varies between studies [16]. Data from large prospective cohort studies, starting before surgery and continuing till a subsequent pregnancy, would be beneficial to obtain insight on reproductive issues following bariatric surgery and to optimize the health care of this specific population.

As a result, main knowledge gaps in the field are related to 1) effective contraceptive counseling, 2) the optimal screening and supplementation strategy for micronutrient deficiencies after surgery before and during a subsequent pregnancy, 3) the best diagnostic method for the diagnosis of gestational diabetes mellitus, and 4) the ideal composition of breast milk from a nutritional point of view, needed before breastfeeding can be safely advised in this sub-population of lactating women.

### Objective and aims

The overall objective of the AURORA study (bAriatric sUrgery Registration in wOmen of Reproductive Age) is to systematically follow women of reproductive age (18–45 year) who will undergo bariatric surgery or who already underwent bariatric surgery until six months after a subsequent pregnancy.

Specific aims are:

1. To evaluate the effect of bariatric surgery in obese women of reproductive age on body composition, nutritional status, fertility, sexuality, contraception, co-morbidities, medication and substance use, physical activity, quality of life, anxiety and depression, by comparing these parameters pre- and postoperatively.
2. To investigate pregnancy outcomes in women with a history of bariatric surgery according to the type of procedure (restrictive, malabsorptive, combined), interval between surgery and conception, weight loss after surgery, nutritional status, gestational weight gain, sexuality, co-morbidities, medication and substance use, physical activity, quality of life, anxiety and depression.
3. To investigate breast milk composition in women with a history of bariatric surgery in relation to the maternal diet and level of physical activity.

## Methods/design

### Overall study design and setting

**Fig. 1:**
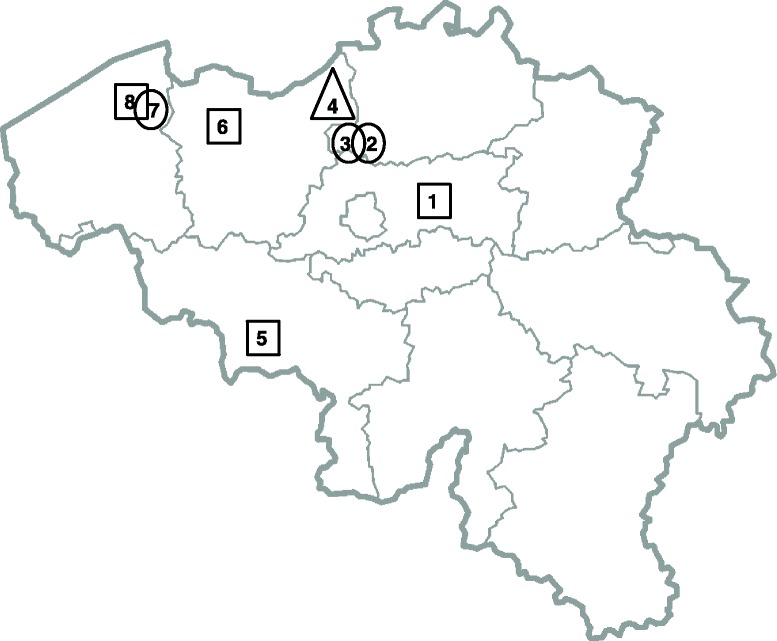
Different sites.  = pregnant and non-pregnant subjects.  = non-pregnant subjects.  = pregnant subjects

AURORA is a national multi-centric prospective cohort study with the participation of eight centers in Belgium: 1) the University Hospital of Leuven (UZL) (=coordinating center), 2) Middelheim Hospital Antwerp (MA), 3) St-Augustinus Hospital Wilrijk (SAW), 4) St-Nikolaas Hospital St-Niklaas (SNSN), 5) Medical Hospital of Mons-Hainaut (CHR), 6) Ghent University Hospital (UZG), 7) St-Jan Hospital Bruges (SJB) and 8) St-Lucas Hospital Bruges (SLB). Figure 1 illustrates the geographical distribution of the participating centers and provides information about the target population for recruitment of each center.

Inclusion is possible at 4 different time points: 1) before surgery, 2) after surgery, 3) at the beginning of pregnancy and 4) in the immediate postpartum (day 3–4). The bariatric surgeon and his team are responsible for the recruitment before and after surgery. Also fertility specialists and obstetricians providing preconception care assist with the recruitment of non-pregnant women with a history of bariatric surgery. Recruitment at the beginning of pregnancy is done by obstetricians and midwifes at the participating centers. Recruitment of lactating women with previous bariatric surgery is done by the researchers/midwifes at the maternity units.

**Fig. 2:**
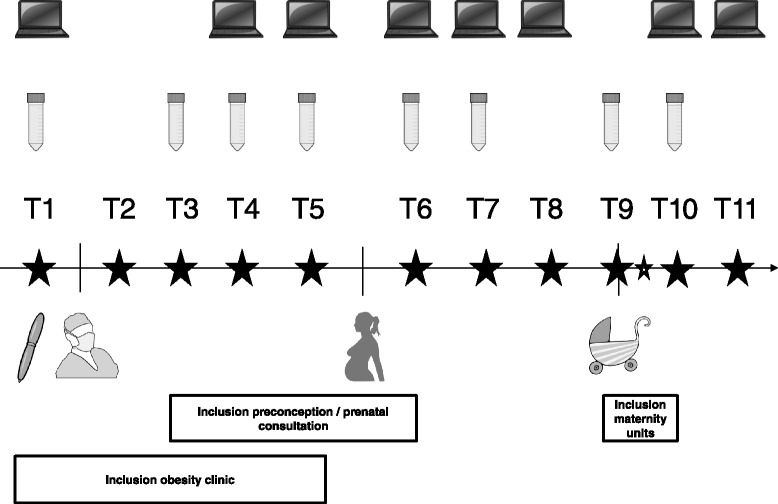
Scheme of measuring moments.  *=* online data collection.  = fasting blood sample.  = bariatric surgery.  = pregnancy.  = birth.  = measuring moment.  = breast milk samples. T1 (before surgery), T2 (3 weeks after surgery), T3 (3 months after surgery, T4 (6 months after surgery), T5 (12 or X months after surgery), T6 (1st trimester of pregnancy), T7 (2nd trimester of pregnancy), T8 (3rd trimester of pregnancy), T9 (birth), T10 (6 weeks postpartum), T11 (6 months postpartum)

The exact scheme of data collection depends on the time of inclusion and is summarized in Fig. 2. A total of five measuring moments is foreseen for a woman being recruited before surgery. Four additional measuring moments are foreseen in a subsequent pregnancy and 2 measuring moments in the postpartum period. A maximum follow-up of 11 measuring points is aimed when recruiting a women before surgery and following her until a subsequent pregnant and six month postpartum period. This equals to a follow-up duration of approximately two and a half year. Women being recruited at the start of pregnancy have a total of six measuring moments, which equals to a follow-up duration of one to one and a half year. Women who gave birth within UZ Leuven are given the opportunity to investigate the macronutrient content of their breast milk. A milk sample of 1.5–2 ml is collected four days after delivery and then weekly until the routine postpartum consultation six weeks after delivery.

### Eligibility criteria

The inclusion and exclusion criteria are summarized in Table 1 and depend on time of inclusion.

**Table 1:** Inclusion and exclusion criteria according to time of inclusion

	Before surgery (T1)	After surgery (T2-T5)	Pregnancy (T6)	Breast milk composition
Inclusion criteria	*women aged 18–45 *approved for bariatric surgery (all types of surgery) *internet connection and email	*women aged 18–45 *previous bariatric surgery (all types of surgery) *internet connection and email	*women aged 18–45 *previous bariatric surgery (all types of surgery) * < 15 weeks of gestation *internet connection and email	*women aged 18–45 *lactating women with previous bariatric surgery *pregnancy duration between 37 and 41 weeks
Exclusion criteria	*infertility (e.g. menopause, hysterectomy)	*infertility (e.g. menopause, hysterectomy)	/	*use of medication possibly affecting breast milk (discussed individually with neonatologist) *problems or difficulties with breastfeeding * > 10 % weight loss in newborn 4 days after delivery *diabetes or GDM

Abbreviations: *GDM* gestational diabetes mellitus, *T* time = measuring moment

### Study visits and data collection

Both clinical and self-administered data are collected before and after surgery and in a subsequent pregnancy and postpartum period. The clinical data are obtained by the local research staff and afterwards directly added in a simple, secure and user-friendly internet database (www.aurorastudy.org/en). Participants receive a link via email to complete the online questionnaires. The system allows participants to log in with their email address, ensuring safe and private access. Participants receive reminders via email when a questionnaire is not completed within one week. Data are encoded directly in the database and transformed to excel for future analyses.

An attempt was made to match the study measuring moments as much as possible with the routine consultations of the participating centers. The data collection at each contact is presented in Table 2.

**Table 2:** Overview of data collection

	T0	T1	T2	T3	T4	T5	T6	T7	T8	T9							T10	T11
											D4	W1	W2	W3	W4	W5	W6	
Researcher																		
Anthropometrics		X	X	X	X	X	X	X	X	X							X	X
Bariatric surgery	X	X	X	X	X	X												
Co-morbidities & medication		X	X	X	X	X	X	X	X								X	X
Blood analysis		X		X	X	X	X	X		X							X	
Pregnancy							X	X	X	X								
Participant																		
Medical & obstetric history	X																	
Demographics	X																	
Lifestyle habits		X			X	X	X	X	X									X
Physical activity		X			X	X	X	X	X		X						X	X
Food Frequency Questionnaire									X		X						X	
3-day food record – dietary supplements		X			X	X	X	X	X									X
Depression							X		X								X	X
Anxiety		X			X	X	X		X								X	X
Quality of life		X			X	X	X		X									X
Intention of becoming pregnant	X^a^																	
Menstrual cycle, contraception, sexuality		X			X	X												
Breastfeeding																		X
Milk sample											X	X	X	X	X	X	X	

Abbreviations: *T0* baseline, *T1* before surgery, *T2* 3 weeks after surgery, *T3* 3 months after surgery, *T4* 6 months after surgery, *T5* 12 or X months after surgery, *T6* 1^st^ trimester of pregnancy, *T7* 2^nd^ trimester of pregnancy, *T8* 3^rd^ trimester of pregnancy, *T9* birth, *T10* 6–8 weeks postpartum, *T11* 6 months postpartum, *D4* day 4 postpartum, *W* 1, 2, 3, 4, 5, 6 weeks postpartum

^a^inclusion pre- and postoperatively

#### Data obtained by the researchers

*Anthropometric data* include body height, weight and fat percentage. The body length is measured at the first measuring moment, using a microtoise to the nearest 0.5 cm. The body weight is measured at each clinical measuring moment to the nearest 0.1 kg with indoor clothing but without shoes, using the local calibrated balance. The pre-operative weight in non-pregnant subjects, together with the subjects’ length, is used to calculate the pre-operative BMI (kg/m^2^). Pregnant subjects were asked for their pre-pregnancy weight (self-administered) to calculate the pre-pregnancy BMI (kg/m^2^). The total gestational weight gain is defined as the weight at time of birth minus the pre-pregnancy weight. The body fat percentage is only measured in non-pregnant subjects, using the local Bio-Electrical Impedance Analysis (BIA).

The m*edical history* of the participant is questioned at baseline (T0), together with the type, date and location of *bariatric surgery*. The medical history relates to the subject’s previous medical conditions and diseases. The specific type of procedure is questioned (LAGB, SG, RYGB and BPD/DS) in order to distinguish between the restrictive, purely malabsorptive or combined types of procedures. The date of the surgery is used to calculate the interval period between surgery and conception.

Follow-up by a dietitian, possible complications, *co-morbidities* and the use of *medication or nutritional supplements* are monitored at each pre- and postoperative and pre- and postnatal follow-up consultation.

The *nutritional status* of each participant is assessed by biochemical parameters in venous blood (Table 3). This screening includes assessments which are covered by the National Institute for Sickness and Disability Insurance (NISDI) as well as specific additional assessments not being covered by the NISDI but covered by study budget. The assessments covered by the NISDI are clinically relevant in the context of an optimal follow-up of the subject after bariatric surgery or during pregnancy. The additional assessments are not included in the standard care, but are considered being relevant as study parameter. The serum blood samples should optimally be collected in a fasting condition. The obesity clinics are responsible for the pre- and postoperative assessments, the obstetric units for the preconception, pre- and postnatal assessments. The nutritional status of the newborn is determined by biochemical parameters in venous cord blood, collected at the delivery wards. These serum markers are analyzed in the local hospitals, all equipped with certified laboratories with validated ring tests.

**Table 3:** Nutritional assessment

	Standard assessment (covered by NISDI/RIZIV)	Additional study assessment (not covered by NISDI – study budget)
Obesity clinic	*haematology (haemoglobin, haematocrit and red blood cell count: mean corpuscular haemoglobin, mean corpuscular volume, mean corpuscular haemoglobin concentration) *coagulation (prothrombin time, activated partial thromboplastin time) *total protein, albumin *vitamin B-12, folate serum *25-hydroxy vitamin D *iron, transferrin, tranferrin saturation, ferritin *calcium, zinc *glucose metabolism (glucose)	*folate red blood cells *vitamin A, E, B-1, magnesium
Obstetrics and gynaecology	*haematology (haemoglobin, haematocrit and red blood cell count: mean corpuscular haemoglobin, mean corpuscular volume, mean corpuscular haemoglobin concentration) *coagulation (prothrombin time, activated partial thromboplastin time) *total protein, albumin *vitamin B-12, folate serum and red blood cells *iron, transferrin, tranferrin saturation, ferritin *calcium, zinc, magnesium *glucose metabolism (glucose)	*coagulation factors, vitamin K1 *vitamin A, vitamin E, vitamin B-1 *25-hydroxy vitamin D
Veneus cord blood		*haematology (haemoglobin, haematocrit and red blood cell count: mean corpuscular haemoglobin, mean corpuscular volume, mean corpuscular haemoglobin concentration) *coagulation (prothrombin time, activated partial thromboplastin time, vitamin K1) *vitamin B-12 and folate serum and red blood cells *vitamin A and 25-hydroxy vitamin D

Abbreviations: *NISDI/RIZIV* National Institute for Health and Disability Insurance/Rijksinstituut voor Ziekte- en Invaliditeitsverzekering

The assessment of the *fasting glucose level* at the beginning and 24 weeks of pregnancy is also included for the pregnant subjects as an alternative for the oral glucose tolerance test (OGTT), which is the standard diagnostic test for GDM [34]. It is well known that a glucose overload in patients with a malabsorptive or mixed procedure surges insulin secretion, probably causing reactive hypoglycemia [22]. The use of an OGTT can therefore be contra-indicated for the diagnosis of GDM in subjects with a history of bariatric surgery. Subjects who report to be sensitive for severe dumping symptoms are screened for GDM by a first and second trimester assessment of the fasting glucose level. When the fasting glucose levels appear to be indicative for a disturbed glucose metabolism, glucose home monitoring will be initiated around 24–28 weeks of gestation. This includes fasting and two hours postprandial glucose monitoring for seven days using a glucose monitoring kit.

*Clinical parameters* of the pregnant subjects are measured by the researcher in every pregnancy trimester (T6-T8). These parameters include gestational age (based on the crown-rump length assessed by fetal ultrasound), blood pressure and proteinuria (>300 mg/24 h). The diagnosis of pregnancy-induced hypertension is defined according to the guidelines of the International Society for the Study of Hypertension in Pregnancy: *de novo* blood pressure ≥ 140/90 mmHg appearing after 20 weeks of gestation. Pre-eclampsia is defined as the presence of pregnancy-induced hypertension or chronic hypertension in combination with proteinuria [35]. All possible complications during pregnancy, delivery and postpartum period are prospectively recorded in the patient’s study file. Next, outcomes from the fetal ultrasounds are summarized (quality of ultrasound, biparietal diameter, abdominal circumference, femur length, head circumference, frontal occipital diameter, estimated fetal weight, amniotic fluid index, pulsatility index of A. Uterina, Umbilicalis and Cerebri Media). In case of a laparoscopic adjustable gastric band, management of the band (closing – opening) is evaluated. The gestational age in weeks, use of combined spinal epidural anesthesia, fetal position and mode of delivery (vaginal, cesarean section, instrumental delivery) are collected at birth (T9). Characteristics from the newborn, including birth weight and length, Apgar scores, admission to the neonatal intensive care unit and clinical problems are reported (T9). Early postpartum data include information about breastfeeding practices (exclusive breast feeding, artificial feeding, difficulties, duration).

Clinical parameters being reported 6 weeks and 6 months after delivery include body weight, the intake of dietary supplements or medication and any clinical problems or complications occurring.

#### Self-administered data by the participants

*Demographic data* are collected at baseline (T0). Date of birth, nationality, ethnicity of the participant and first-line family members, marital status, family situation, educational level and occupational data are questioned.

The *obstetric history* of the participant is also collected at baseline (T0). This includes the number of previous pregnancies, the need for a fertility treatment, the final outcome of a previous pregnancy (miscarriage, abortion, ectopic pregnancy, born alive) and gestational age and birth weight of the baby.

Participants who are included before or after surgery are asked for their *intention of becoming pregnant* and the period in which they plan to do this.

A study-specific developed questionnaire is used to evaluate the *menstrual cycle, contraceptive use and sexual activity.* The length of the menstrual cycle, the cycles per year, pattern and duration of bleedings and the presence of amenorrhea or oligomenorrhea are questioned. The need for a fertility treatment and the method of the treatment is asked. The used contraceptive is also of interest, including the exact type, dose and problems with usage. The frequency of sexual intercourse and any related problems are recorded. These questionnaires are based on the standard clinical assessments at the gynecology clinic of UZ Leuven.

*Lifestyle* is being investigated using questionnaires about dietary intake, physical activity, alcohol, tobacco use and sleeping habits, all collected via the online system. The *actual diet* is examined on three non-consecutive days (including one day of the weekend) by use of a 3-day estimated dietary record, using an online semi-structured diary. Information on the type and amount of foods consumed is collected through an open entry format. Each day of the food record was divided into breakfast, lunch, dinner and morning, afternoon and late-evening snacks. Subjects who donate breast milk samples are additionally asked to complete a Food Frequency Questionnaire (FFQ) in the immediate postpartum (day 3–4) and 6 weeks postpartum. This FFQ is inspired on pre-existing FFQ’s already used in the Belgian population groups (e.g. [36]) and on the general accepted FFQ development guidelines [37]. The FFQ allows to monitor trends in nutrient intake and dietary pattern (=*usual intake*). The level of *physical activity* is measured using a Dutch and French version of the Kaiser Physical Activity Survey (KPAS). This survey measures different domains of physical activity including 1) household and family care, 2) professional activities, 3) daily life activities and 4) sports and exercise. The KPAS is validated for both pregnant and non-pregnant women [38, 39]. A Dutch and French version of the Alcohol Abuse Disorder Identification Test (AUDIT-C) includes three short questions on *alcohol use* and is considered to be a practical and validated screening test to detect alcohol abuse and dependency [40]. S*moking* is questioned, and for smokers the amount of cigarettes or other tobacco products per day is recorded. Finally, sleep duration during the week and week-end is questioned.

The intake of *dietary supplements* is also questioned via an online questionnaire (brand name, dosage, posology, start and end date).

The *quality of life* is determined using the Moorehead-Ardelt Quality of Life Questionnaire II (M-A QoLQII) [41]. This validated questionnaire is part of the Bariatric Analysis and Reporting Outcome System (BAROS) [42]. BAROS is a scoring system to evaluate quality of life (M-A QoLQII), weight loss and improvement of co-morbidities. The M-A-QoLQII is translated in Dutch by two experts [43] and in French by the research team.

Evaluation of *psychosocial health* focuses on anxiety and depression. A Dutch validated and a French non-validated version of the American State-Trait Anxiety Inventory evaluates the two concepts of ‘state and trait anxiety’ [44]. Two separate questionnaires include 20 items or statements on which answers can be given on an intensity (state anxiety) or frequency (trait anxiety) scale. A Dutch and French version of the Edinburgh Depression Scale (EDS), a validated 10-item scale, is used to measure depressive feelings during the pre- and postnatal period [45–47].

Finally, a structured Dutch questionnaire developed by Guelinckx et al. (2011), also translated into French by the researchers, is used six months after birth (T11) to evaluate the intention, initiation and duration of breastfeeding and reasons to quit breastfeeding.

#### Analysis of the breast milk macronutrient composition

Human milk samples of 1.5 to 2 ml are collected from subjects who gave birth at UZ Leuven. A first sample is being collected at day 4 after delivery at the maternity units. Samples are collected manually or by use of a pump three minutes after the baby starts suckling on the breast. Samples are stored at−20 °C [48]. Subjects receive instructions by the researchers on how to collect a weekly sample by themselves at home until the routine consultation six weeks after delivery. They receive sterile recipients to collect the samples and a thermometer to measure the temperature of their own freezers. All samples are gathered at the research center when all samples are collected six weeks after birth.

The human milk macronutrient (fat, carbohydrate, protein) and energy content is easily assessed by use of the MIRIS® Human Milk Analyzer. This is a relatively small device that is based on infrared spectroscopy, and requires very limited amounts of milk (1.5 to 2 ml) [49]. The iCheck™ Fluoro (BioAnalyt, Germany) is used for the analysis of the vitamin A content in breast milk. This is also a small and user-friendly device that is validated against High-Performance Liquid Chromatography (HPLC) which is the reference method for vitamin A analysis in breast milk [50].

### Type of analyses

The study design allows different types of analytical procedures, including descriptive analyses, cohort study analyses and even case–control study analyses as our study team is also focusing on obese (pregnant) women in other projects. Analyses will be performed using SPSS software and a specific *P*-value of significance will be determined based on the number of test that will be performed for a specific research question. The normality of continuous variables will be checked with normality tests (Kolmogorov-Smirnov or Shapiro Wilk’s test). Depending on the normality outcomes, parametric (Student-t, one-way AN(C)OVA, repeated measures AN(C)OVA, Chi^2^, Pearson, multiple and logistic regression test) or non-parametric (Mann–Whitney U, Kruskall Wallis, Spearman and Friedman tests) tests will be used. Interaction, group (different types of surgery, pregnant ≤ or > one year following surgery, bariatric surgery versus obese and normal weight controls, …) and time (before surgery versus 6 and 12 months after surgery, before pregnancy versus different pregnancy trimesters, pre- versus postnatal, …) effects will be comprehensively examined. A biostatician will be consulted to assist with the statistical analyses.

### Power calculation

No power calculation has been performed for the prospective cohort study because of the many potential outcome measures with varying incidence numbers. The aim is to include as many women as possible, starting pre- or postoperatively and in a subsequent pregnancy We intend to expand the group of non-pregnant women to at least 300 women at the end of 2016 and the group of women being included at the start of pregnancy to a total of 150 women. This appears to be realistic based on the current clinical experience.

A power calculation was performed for the breast milk macronutrient analyses. Based on the study of Marin et al. (2005) [51], it was calculated how many subjects are needed in a group of lactating women with a history of bariatric surgery and in obese, overweight and normal weight controls. A difference of 10 g/L in fat content between normal and obese mothers and a standard deviation of 9.4 g/L, together with a sample size power of 90 %, were used for the sample size calculation. When taking into consideration a 30 % drop-out rate, a total of 72 women (24 in each group) is needed.

### Start of the study and expected results

Recruitment of the first participant took place in December 2012. First research papers on various topics within this study are expected to be published in 2016.

## Discussion

According to the authors’ knowledge, AURORA is the first prospective cohort study which starts to include women undergoing a bariatric procedure until a subsequent pregnancy and postpartum period. Its strength is the long duration of follow-up and the unique collaboration between obesity and obstetric units. A large number of females who underwent bariatric surgery, both pregnant and non-pregnant, can be achieved as a result of the multi-center approach. The online secured database allows easy gathering of clinical data obtained by the researchers across the different research sites, and provides an easy manner of data collection from participants. The online system is also accompanied with limitations. The more non-directive approach of participants might probably lead to a higher rate of missing values when comparing to the standard approach of distributing paper questionnaires personally to the subjects, and to a relatively large drop-out rate during follow-up. This rate is currently estimated around 15 %. Since this study relies on online data gathering, a temporary failure of the system could mean a temporary shutdown of data collection. For this reason, paper case report forms (CRFs) were designed and spread across the different research sites. Local researchers and their staff are asked to complete a CRF for each visit of a patient which equals to a measuring moment in order to serve as back-up. For participants, this would imply temporary inconvenience and data loss.

The greatest challenge is to obtain the complete follow-up of patients being recruited before surgery until a subsequent pregnancy. Official organizations recommend to postpone pregnancy until 12 to 18 months after surgery to avoid the phase of rapid weight loss [6]. When taking into consideration the minimal time interval between surgery and conception of 12 months, and assuming there are less to none fertility problems, a total follow-up will take about two and a half year. Participants are reminded every six months of possible continuation of the study when they should become pregnant. So far, this seems to be sufficient in order to ensure that no pregnancies are missed among women being recruited before or after surgery. It is estimated that about 12 000 bariatric procedures are being performed per year in Belgium [8]. If we assume that Belgium follows to international trends of growing numbers of female adolescents attending for bariatric surgery and the majority of bariatric patients being premenopausal women (about 70 %) [12, 13], we can assume that 300 is a realistic number We aim to include sufficient lactating subjects with a history of bariatric surgery. It is demonstrated that obese women are less likely to initiate and continue with breastfeeding [5], and so far, no breastfeeding rates are known of lactating women with previous bariatric surgery. Depending on the number of inclusions so far, recruitment of lactating women with previous bariatric surgery has additionally started in other local obstetric centers within AURORA (UZG and SAW). Efforts to motivate and stimulate participants to collect breast milk samples are made by weekly text messages, emails or phone calls, depending on the participant’s preference.

Another discussion point relates to the local devices used for anthropometric measurements. The fact that the local scales and bio-impedance devices are used, might lead to a certain degree of bias. This remark also relates to the nutritional biochemical assessments. Serum markers are analyzed in the local hospitals, all with certified laboratories with validated ring tests. Nevertheless, small differences in analyses, especially for the more complex assessments of fat-soluble vitamins and coagulation markers, may implicate small differences in outcome values. Finally, no specific actions are undertaken to include women from all socio-economic backgrounds. This may lead to the inclusion of more women with a specific income and educational level, which in turn might lead to less generalization of the outcomes for follow-up and lifestyle.

The AURORA study also has strong potential to clarify the combined impact of bariatric surgery and a subsequent pregnancy on maternal, fetal and neonatal health. The study comprises a combination of many medical and personal data of pregnant and non-pregnant women with bariatric surgery. The study includes regular and intensive monitoring of nutritional biochemical parameters, combined in pregnancy with the assessment of fetal growth by ultrasound, the monitoring of maternal weight, the dietary assessment (of dietary intake) and intake of dietary supplements. Prevalence and incidence number of nutritional deficiencies, surgical complications and more uncommon outcomes such as birth defects can be evaluated within this cohort.

The findings from the AURORA study are believed to generate a significant public health impact. Outcomes obtained from this study will be translated into clinical guidelines and recommendations for the reproductive management of women undergoing bariatric surgery and subsequently becoming pregnant.

## Abbreviations

AUDIT-C, alcohol abuse disorder identification test; BAROS, bariatric analysis and reporting outcome system; BIA, bio-electrical impedance analysis; BMI, body mass index; BPD/DS, biliopancreatic diversion with or without duodenal switch; CRF(s), case report form(s); CS, cesarean section; EDS, Edinburgh depression scale; KPAS, Kaiser physical activity survey; LAGB, laparoscopic adjustable gastric banding; M-A QoLQII, Moorehead-Ardelt Quality of Life Questionnaire II; PIH, pregnancy induced hypertension; RYGB, roux-en-y gastric bypass; SG, sleeve gastrectomy; WHO, World Health Organization.
